# eRNA: a graphic user interface-based tool optimized for large data analysis from high-throughput RNA sequencing

**DOI:** 10.1186/1471-2164-15-176

**Published:** 2014-03-05

**Authors:** Tiezheng Yuan, Xiaoyi Huang, Rachel L Dittmar, Meijun Du, Manish Kohli, Lisa Boardman, Stephen N Thibodeau, Liang Wang

**Affiliations:** 1 Department of Pathology and MCW Cancer Center, Medical College of Wisconsin, Milwaukee WI 53226, USA; 2 Department of Oncology, Mayo Clinic, Rochester MN 55905, USA; 3 Department of Laboratory Medicine and Pathology, Mayo Clinic, Rochester MN 55905, USA

**Keywords:** RNA sequencing, Bioinformatics tool, Graphic user interface, Parallel processing

## Abstract

**Background:**

RNA sequencing (RNA-seq) is emerging as a critical approach in biological research. However, its high-throughput advantage is significantly limited by the capacity of bioinformatics tools. The research community urgently needs user-friendly tools to efficiently analyze the complicated data generated by high throughput sequencers.

**Results:**

We developed a standalone tool with graphic user interface (GUI)-based analytic modules, known as eRNA. The capacity of performing parallel processing and sample management facilitates large data analyses by maximizing hardware usage and freeing users from tediously handling sequencing data. The module miRNA identification” includes GUIs for raw data reading, adapter removal, sequence alignment, and read counting. The module “mRNA identification” includes GUIs for reference sequences, genome mapping, transcript assembling, and differential expression. The module “Target screening” provides expression profiling analyses and graphic visualization. The module “Self-testing” offers the directory setups, sample management, and a check for third-party package dependency. Integration of other GUIs including Bowtie, miRDeep2, and miRspring extend the program’s functionality.

**Conclusions:**

eRNA focuses on the common tools required for the mapping and quantification analysis of miRNA-seq and mRNA-seq data. The software package provides an additional choice for scientists who require a user-friendly computing environment and high-throughput capacity for large data analysis. eRNA is available for free download at https://sourceforge.net/projects/erna/?source=directory.

## Background

Advances in high-throughput sequencing (HTS) technologies have achieved the analysis of genome-wide RNA profiles with high accuracy and unprecedentedly deep coverage while costs continue to decrease. The Illumina Hiseq 2500 sequencing system is able to sequence 192 RNA samples (multiplexed 24 samples in a single lane) up to six billion paired-end reads in a run (http://support.illumina.com/). Due to its high capacity, RNA sequencing (RNA-seq) has become a necessary research approach for transcriptomic studies and integrated systems analyses.

To date, many bioinformatics tools have been developed to support the identification of known RNAs and analysis of RNA expression profiles. A common workflow for micro-RNA sequencing (miRNA-seq) analysis includes adapter removal, sequence alignment, and read counting. To complete this process, various tools have been developed, including DSAP [1], E-miR [2], miRanalyzer [3], miRDeep2 [4], MIReNA [5], miRExpress [6], miRNAkey [7], miRspring [8], mirTools [9], and SeqBuster [10] (Additional file 1: Table S1). These miRNA tools perform very well with respect to sensitivity, accuracy, and visualization for miRNA identification [11]. Unlike miRNA-seq, a popular workflow for mRNA sequencing (mRNA-seq) analysis includes genome mapping, transcript assembling, and differential expression analysis, each separately accomplished by a combination of standalone tools (namely a combination of Bowtie, SAMtools, TopHat, and Cufflinks) and R packages in R environments [12]. Some open source analytic workbenches or software solutions have been developed to integrate these different third-party tools, such as ArrayExpressHTS [13], Chipster [14], ExpressionPlot [15], GENE-Counter [16], GenePattern (http://www.broadinstitute.org/cancer/software/genepattern/modules/RNA-seq), GeneProf [17], RNA-seq Toolkit (RST) [18], RobiNA [19], and TCW [20] (Additional file 1: Table S2). Of these, the web-based tools provide a GUI-based computer platform. User friendly access to web browsers makes RNA-seq data analysis possible for broad research scientists. The standalone tools, however, are more flexible than the web-based tools. Due to local installation and operation, users may adjust the parameters or even write a program using command codes to meet their specific requirements. For some open-source tools, users may revise the codes and integrate them into their own workflow for RNA-seq data analysis.

Although there are few limits on sequencing data outputs and sample sizes, the use of current bioinformatics tools remains challenging for broad research scientists due to insufficient abilities to process large data, as well as the limitation on data inputs and sample management. Large data analysis and multiple RNA sample management through the web-based tools are not practical due to the limits of network connection, the ability of server computers, and the security of remote data storage. It is also time-consuming to upload sequencing data and reference sequences. In some cases, additional modifications are required prior to miRNA analysis. For example, users have to trim adapter sequences and convert the inputs from FASTQ to FASTA format when using some miRNA tools (namely mirTools or miRspring) [21]. The lack of sample management further complicates data analysis and increases the potential for errors. It is impossible to analyze a large data set from numerous biological samples with different traits along with their technical and biological replicates when only one RNA sample can be processed at a time. Furthermore, computation running time under large data processing is another challenge for RNA-seq data analysis. Some tools process the datasets from only one RNA sample at a time because of their limits on parallel processing. In addition, for scientists without any programming experience, it is often difficult to perform parameter setting and data format converting in command-line tools. Although some standalone tools are user-friendly for bioinformaticians and computer scientists, mastering such knowledge is often beyond the comfort level for most research scientists. To meet these challenges, we developed a GUI-based tool called eRNA, which integrates common tools required for RNA-seq analysis and facilitates large-scale data analysis.

## Implementation

**Figure 1:**
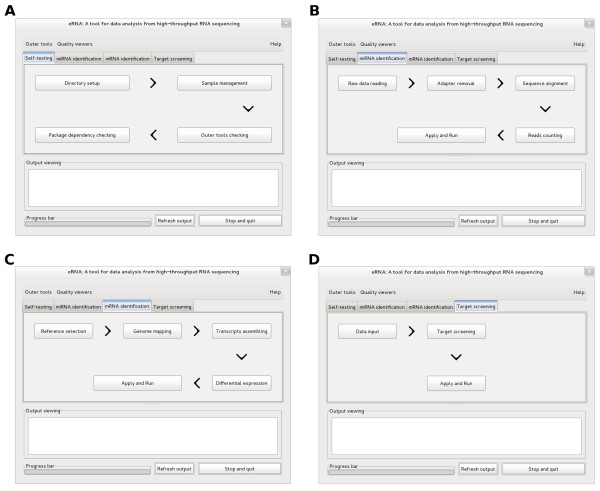
**The modules in eRNA. (A)** The module “Self-testing”. **(B)** The module “miRNA identification”. **(C)** The module “mRNA identification”. **(D)** The module ”Target screening”.

eRNA can be operated in a user friendly running environment. eRNA’s interface has a main cascade graphic user interface (GUI) where multiple button operations trigger three-cascade sub-windows. All of the operations for each module are accompanied by step-by-step guides, and all parameters required for data analysis can be set through the GUIs. eRNA is divided into several functional GUI-based modules, which can be flexibly used in any combination or separately operated based on the requirements for data analysis. The modules “Self-testing”, “miRNA identification”, “mRNA identification”, and “Target screening” are presented as notebook pages in the main graphic interface (Figure 1). After successfully operating “Self-testing”, users can easily follow the parameter setup guided by the arrow from left to right within notebook pages to perform data analysis of miRNA identification, mRNA identification, or RNA expression profiles. This design allows a new user to start RNA-seq data analysis with minimal self-teaching. eRNA’s fast parameter setup and error-free input format are superior to command-line tools. Due to the long duration of data analysis, a visualization bar is added to show the program running status, and at the same time a monitoring GUI is also activated to show the analytic status of each sample as well as the computer system load. eRNA also supports a refreshing run after the first-time setup to save time. Users can easily revise any parameters in a certain step based on previous results, and the refreshing run will not change the results determined by previous steps in the eRNA’s pipeline. If a computer lacks support for Perl-Gtk2, eRNA can be operated by command. Users can revise all variables in the parameters file “variables.txt” (which is always in the result directory set by users) in command-line mode, of which parameter setup, especially in the third-party tools integrated in eRNA, is more flexible.

### Self-testing

This is the initial step in the RNA-seq analysis pipeline and should be performed before any other modules. This module guides all analytic steps for a successful run in eRNA, including directory setup, sample management, third-party tools checking, and package dependency checking (Figure 1A). The directory setup allows re-allocation of raw data and results in more than one hard drive in a computer. Sample management is used for task assignment in parallel processing and creating associations among raw data, RNA samples, and the biological traits. The third-party tools and package dependency checks are used for detection of third-party RNA analytic tools and Perl packages required by eRNA. With raw data in FASTQ format and reference sequences in FASTA format as data input, the eRNA software package integrated with the third-party tools is able to perform miRNA or mRNA-seq data analysis.

### miRNA identification

**Figure 2:**
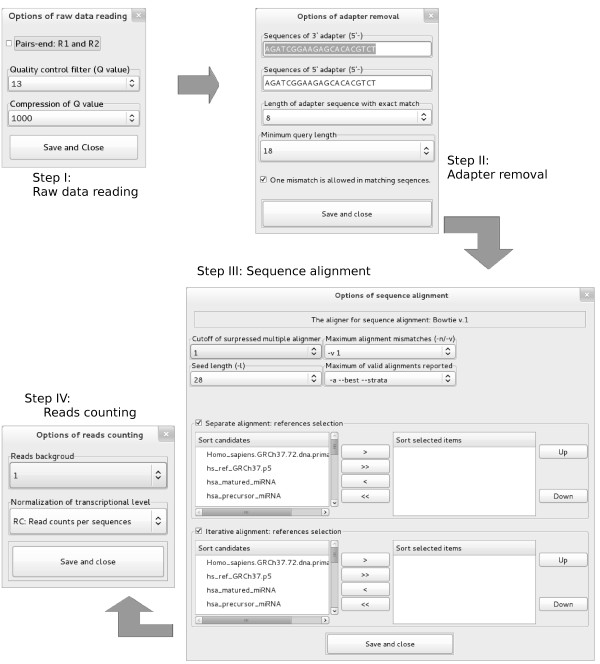
The GUIs of the module “miRNA identification”.

The pipeline in this module (Figure 1B) is categorized into “Raw data reading”, “Adapter removal”, “Sequence alignment”, and “Reads counting” as a GUI-based step by step approach (Figure 2). A third-party aligner (the default is Bowtie1) is required in this module [22]. The accurate identification of mature miRNAs based on their sequences alone is often difficult because miRNAs are short (21-23 nt) and sometimes have similar or even identical sequences. For example, has-miR-519c-5p and has-miR-523-5p have the same sequence and members of the let-7 family have similar sequences. Several miRNA families (e.g., hsa-let-7 and hsa-miR-30) consist of highly homologous miRNAs that differ by only a single or a few nucleotides. Sequence alignment in eRNA therefore includes the methods of separate and iterative alignments (the panel “Step III” in Figure 2). The separate alignment option aligns all sequences against different references separately in a run. The iterative alignment option aligns sequences against the reference sequences in a pre-determined order. The unmapped sequences in previous alignments will be used as query sequences in the next alignment. Besides the setting parameters of the aligner, the sequential order of references, in particular those with closely related sequences, may affect the final result in the iterative alignment. For example, the final results will be significantly affected when mapping precursors and mature miRNA are in a different order.

### mRNA identification

**Figure 3:**
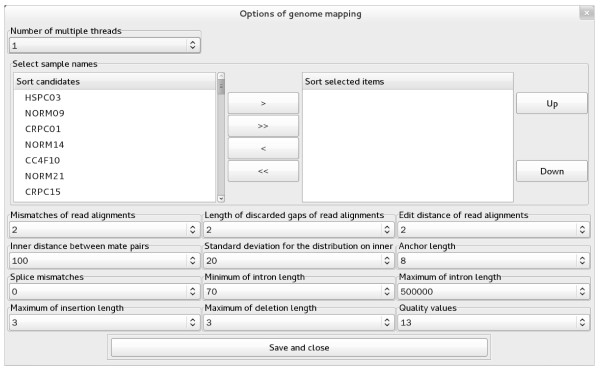
The GUI of TopHat for genome mapping in the module “mRNA identification”.

**Figure 4:**
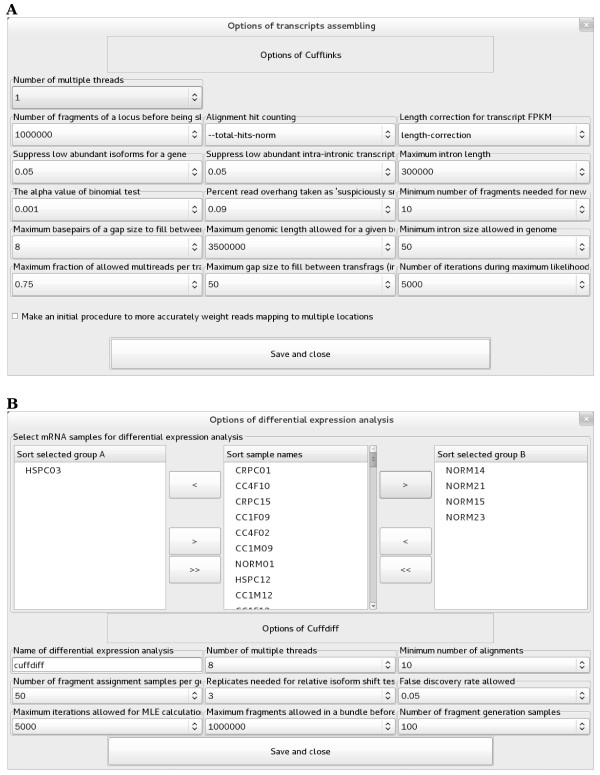
**The GUIs of Cufflinks in the module "mRNA identification". (A)** Transcript assembling. **(B)** Differential expression profiling analysis.

The pipeline in this module is categorized into “Reference sequences”, “Genome mapping”, “Transcripts assembling”, and “Differential expression” (Figure 1C). Except for the RNA sample selection, all parameters presented in the GUIs are the same as those in TopHat [23] and Cufflinks [24]. Due to GUI-based parameter setup and RNA sample selection, eRNA requires less time than command line-based TopHat (Figure 3) and Cufflinks (Figure 4). In addition, eRNA allows for parallel processing to maximize computation capacity, which is different from the serial runs of those commands provided by TopHat and Cufflinks.

### Target screening

This module performs differential expression profiling analysis and recursive partitioning analysis (Figure 1D). R environment and R packages are required for this module. The former pipeline utilizes the method implemented in the R package DESeq to reveal differential expressed genes between two groups of given RNA samples [25]. The latter pipeline utilizes the model implemented in the R package “Party” to predict the importance of expressed genes determined by the modules known as miRNA or mRNA identification dependent on the biological traits within the given RNA samples [26].

In summary, raw data and reference sequence preparation, sample information input, and software parameter settings in eRNA are optimized to ensure a user friendly environment. The learning time to understand RNA data analysis is minimized. The preparation of raw data and references in a successful run is significantly simplified.

## Results

### Sample management

**Figure 5:**
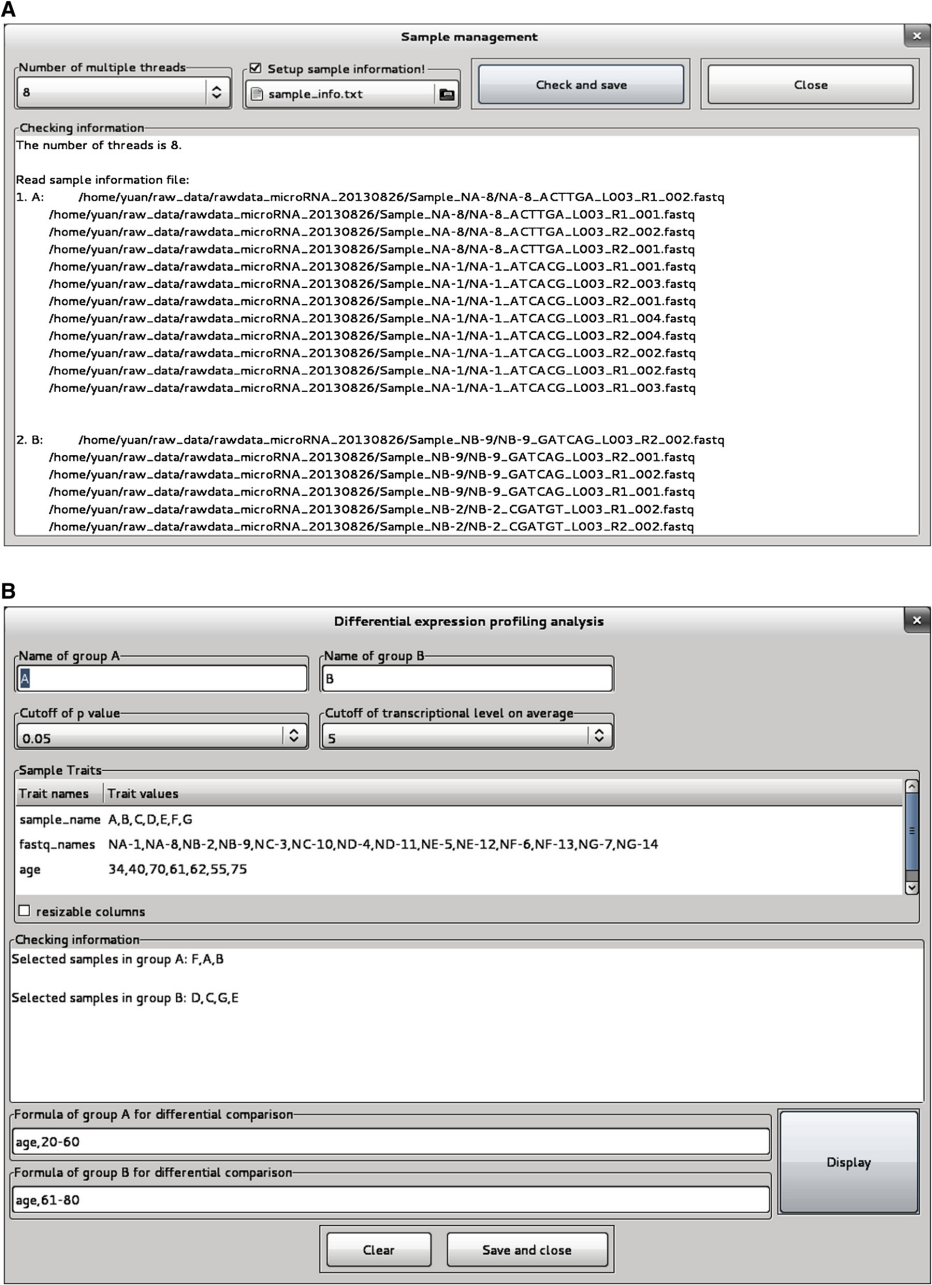
**Sample management in program mode in eRNA. (A)** Automatic connections between miRNA samples (sample names) and raw data (FASTQ format files). **(B)** Automatic connections between miRNA samples and the traits of miRNA samples for differential expression analysis.

eRNA can automatically establish the connections among RNA samples, raw data (FASTQ format files), and the traits of RNA samples (Figure 5). In auto mode, eRNA recognizes raw data in a certain directory and automatically associates them with RNA samples. The association rule between raw data and RNA samples is based on the names of the FASTQ files. This association has no limit on raw data size and is able to combine separate FASTQ files for specific RNA samples and identify group relationships among RNA samples. Therefore, the process of data input is simplified at the FASTQ file level, different from the one-by-one data input offered by other tools. In program mode, eRNA automatically connects RNA samples with raw data based on a text file containing pre-annotated RNA samples (Figure 5A). Program mode includes all functions in auto mode and more specific functions for customized applications. For example, program mode allows data combination of biological replicates with the same names or different RNA samples. This mode can also automatically establish connections between RNA samples and their traits. Therefore, RNA samples can be quickly selected among a large number of samples for expression profiling analysis in the target screening module (Figure 5B).

Another feature of sample management in eRNA is the distribution of the data flow in parallel processing. Once parallel analysis is triggered, the whole analytic work is split into certain components consistent with the number of multi-threads. eRNA automatically distributes the raw data to different components as the inputs based on the size of raw data for each RNA sample. The data in each component are analyzed separately and simultaneously.

### Case study on miRNA-seq data analysis

**Figure 6:**
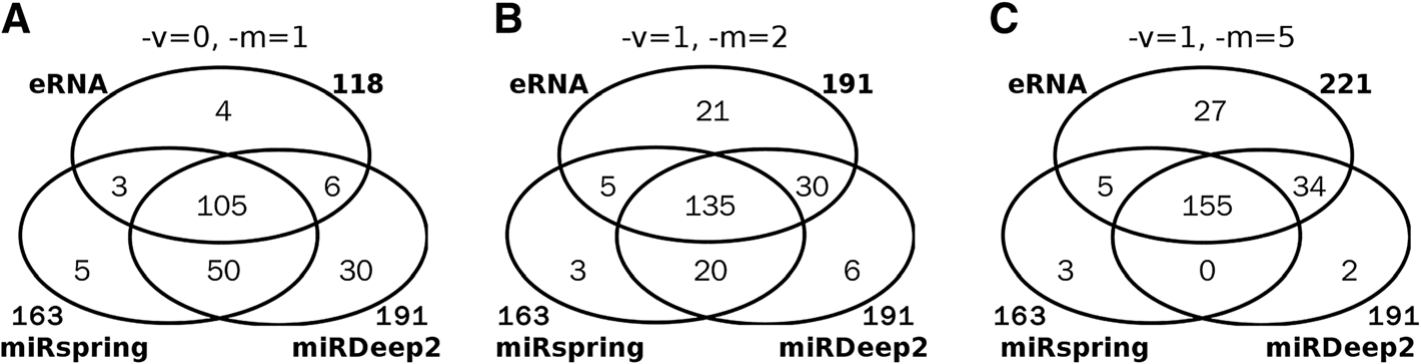
**Venn diagram of eRNA, miRspring and miRDeep2 on known miRNA precursors identification.** Options of Bowtie1 used in eRNA: **(A)** -v 0 -m 1 -a - -best - -strata, **(B)** -v 1 -m 2 -a - -best - -strata, **(C)** -v 1 -m 5 -a - -best - -strata. Default options from Bowtie1 used in miRspring: -v 1 -a - -best - -strata. Default options from Bowtie1 used in miRdeep2: -v 1 -a - -best - -strata - -norc. -v: the maximum number of mismatches in the report alignments. -m: the maximum number of the suppressed alignments if a read has multiple reportable alignments. -a - -best - -strata: Bowtie reports only those alignments in the best stratum.

**Figure 7:**
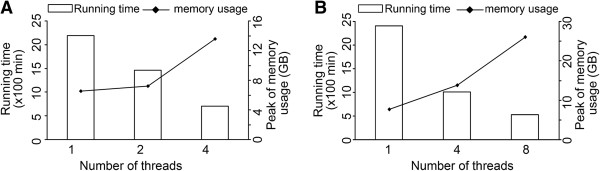
**Correlation between the increased number of multi-threads for miRNA-seq data analysis and decreased running times. (A)** The GUI mode in the personal computer (1 CPU). **(B)** Command line mode in the server computer (4 CPUs).

To evaluate the performance of eRNA on miRNA identification, seven miRNA samples and their replicates were extracted from plasma exosomes of 7 human participants. The participants gave written informed consent for the use of their tissue samples for this study. Exosome isolation, RNA extraction, and miRNA library preparation have been previously reported [27]. These samples were sequenced using an Illumina Hiseq 2000 DNA sequencing analyzer. Raw data can be downloaded from http://www.ncbi.nlm.nih.gov/geo/query/acc.cgi?acc=GSE53451. Human miRNA sequences were downloaded from miRBase (http://www.mirbase.org/, Release 19, 2,043 entries) [28]. Human genome sequences were downloaded from the NCBI file transfer protocol (FTP) site (ftp://ftp.ncbi.nlm.nih.gov/genomes/H_sapiens/, 2 November 2012, Release 104), using the assembly build GRCh37.p10. Bowtie1 (version 0.12.8) was used for sequence alignment [22]. Identification of known matured miRNAs determined by eRNA was compared with the results determined by miRDeep2 [4] and miRspring [8]. Our study showed that there was no significant difference between identified known miRNAs among eRNA, miRspring, and miRDeep2 based on the same raw data of miRNA-seq, sequence aligner (Bowtie1), and miRNA references sequences (Figure 6). However, with increase of mismatches (-v) and multiple alignments (-m) in Bowtie1 options, miRNA precursors identified by eRNA covered almost all precursors identified by miRspring and miRDeep2, due to the different considerations of these tools on multiple or non-exactly matching alignments. To improve the ability of eRNA with large miRNA data analysis, we applied multi-threads technology, which assigns CPU sources (in a computer with multiple CPUs or CPU cores) to different analytic channels for parallel data analysis. Multi-threads processing in eRNA can achieve the optimized balance between the ability of computer hardware and the amount of miRNA-seq or mRNA-seq data. The results showed that computation time decreased as the number of threads increased either in a personal computer (Figure 7A) or in a server computer (Figure 7B); the peaks of memory usages per GB data in both testing environments are uniform.

### The case study on mRNA-seq data analysis

**Figure 8:**
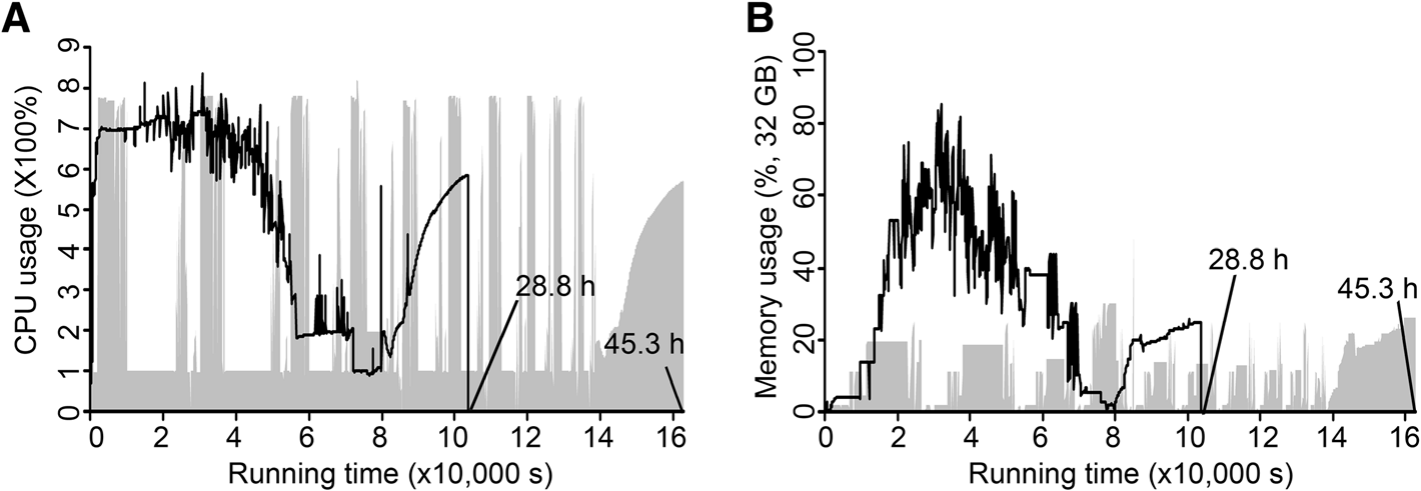
**Running time under various CPU usages (A) and memory usages (B) for mRNA-seq data analysis.** The area under the solid line shows the changes in running time, CPU and memory usage when multithreading is activated only in TopHat and Cufflinks. The grey area shows their changes when the multithreading is activated only in eRNA.

To test the capability of eRNA for mRNA data analysis, ten mRNA samples were extracted from normal human prostate tissue samples and sequenced. The participants gave written informed consent for the use of their tissue samples for this study. The use of these bio-specimens was approved by the Institutional Review Boards at the Medical College of Wisconsin, Milwaukee, WI, and Mayo Clinic, Rochester, MN. Raw data can be downloaded from http://www.ncbi.nlm.nih.gov/geo/query/acc.cgi?acc=GSE53452. The whole mRNA-seq analysis process was finished by eRNA integrated with the third-party tools Bowtie2 (version 2.1.0) [29], SAMtools (version, 0.1.19) [30], TopHat (version 2.0.8) [23], and Cufflinks (version 2.1.1) [24]. The use of GUIs for the parameter settings in eRNA is more intuitive than the long laborious command line arguments in TopHat and Cufflinks. Furthermore, eRNA has optimized the use of multi-threading in mRNA-seq data analysis. Similar to TopHat, Bowtie and Cufflinks, it also takes advantage of multi-threads to speed up mRNA-seq data analysis. However, eRNA applies multi-threads to the entire analytic process, differing from the utilization of multi-threads in partial steps in those tools. Within the maximum-allowed system load and memory usage of the computers (the highest number of multi-threads is eight), the running time declined 36% from 45.3 hours to 28.8 hours at the cost of high efficient CPU usage (Figure 8A) and memory usage (Figure 8B) after multi-threads optimization in eRNA (the number of multi-threads in eRNA, TopHat and Cufflinks are 8:1:1) when compared to memory usage without multi-threads optimization (the number of multi-threads in eRNA, TopHat and Cufflinks are 1:8:8). This result reveals that multi-threads utilization in eRNA can expedite mRNA analysis during parallel processing, compared with separate runs of these third-party tools (TopHat, Bowtie, and Cufflinks).

### Function extension

**Figure 9:**
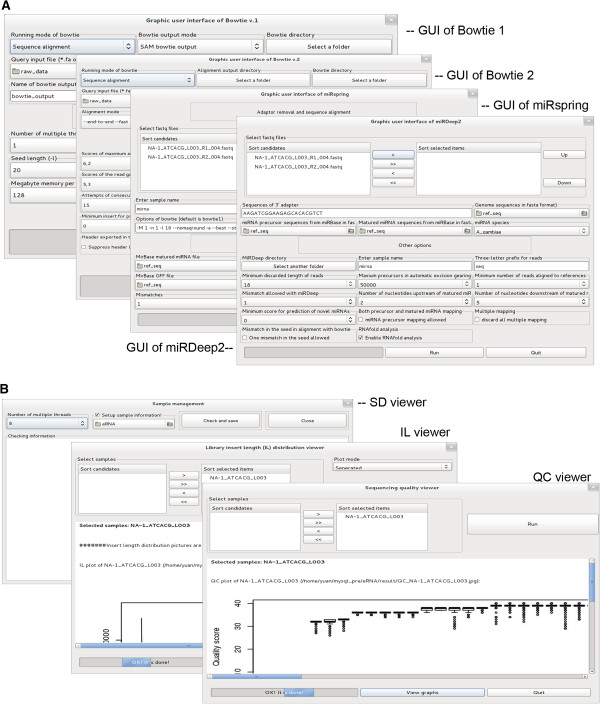
**Plug-in tools for eRNA. (A)** GUI of the third-party tools. **(B)** Graphic viewers for quality control.

To extend the applications of eRNA, we developed plug-in tools, which can be run independently from the modules of eRNA. Of these plug-in tools, GUIs for third-party tools are listed in the menu “Edit”, and graphic viewers for sequencing quality control are listed in the menu “View” (Figure 9).

### GUIs of the aligners

The GUIs allow users to apply the aligners Bowtie1 [22] and Bowtie2 [29] for sequence alignment, including index building separately from the other pipelines provided by eRNA (Figure 9A). Fourteen of 64 optional parameters in Bowtie (v.1) are involved in the Bowtie1 GUI and 22 of 73 of Bowtie (v.2) are involved in the Bowtie2 GUI.

### GUIs of the third-party miRNA tools

miRspring [8] and miRDeep2 [4] GUI along with the miRNA module provide common tools for miRNA seq analysis (Table 1). Operations through the miRspring and miRDeep2 GUI are more user-friendly and less restrictive than the operations of these miRNA tools through command lines (Figure 9A). miRspring is good at visualization, calculation, and reporting on the complexities of miRNA processing. However, it does not support FASTQ format inputs, adapter removal, and sequence alignment, which must be finished in advance. The miRspring GUI in eRNA supports raw data input and provides the whole pipeline of miRNA-seq data analysis including the pipelines finished by miRspring and the pipeline of raw data reading, adapter removal, and sequence alignment finished by eRNA. miRDeep2 is designed for the identification and discovery of known and novel miRNA genes. It also improves the identification algorithms of canonical and non-canonical miRNAs. The miRDeep2 GUI in eRNA supports all operations provided by miRDeep2 and simplifies raw data input, adapter removal, and miRNA reference sequence preparation.

**Table 1:** Functional comparison of eRNA, miRspring, and miRDeep2

**Functionality**	**eRNA**	**miRspring**	**miRDeep2**
Identification of known miRNAs	**+**	**+**	**+**
Visualization of analytic results	**-**	**+**	**+**
Discovery of miRNAs	**-**	**-**	**+**
miRNA expression profiling analysis	**+**	**-**	**+**
Batch data processing	**+**	**-**	**-**
Visualization of sequencing quality control	**+**	**-**	**-**

+, available functions. -, unavailable functions.

### Graphic viewers for quality control

Graphic viewers known as QS Viewer, SD Viewer, and IL Viewer are used for sequencing quality control in RNA-seq experiments (Figure 9B). QS Viewer can plot distributions of quality scores (Q score) per sequencing cycle for each miRNA sample, which can be used for sequencing quality testing [21]. SD Viewer can plot RNAs against certain reference sequences to display sequencing depth, indicating transcript abundance. IL Viewer can plot the distribution of insert lengths from the sequencing library to show the general quality of RNA sequencing library construction.

## Discussion

It is challenging for developers to strike a balance between a user-friendly environment and high efficiency with respect to the processing of RNA-seq data analysis. GUIs and sample management in eRNA provide a user-friendly environment and fulfill the requirements for large data analysis. The use of multi-threads technology makes parallel processing of RNA-seq data possible. The objectives of eRNA are listed as follows:

### Low dependency

As a rule, such a tool should be easy to use and require no prior knowledge of specific computer programming language. GUIs will save time in learning how to use this software. A user-friendly framework will allow biological researchers to focus on RNA data analysis and biological interpretation. Also, preparations of raw data and reference sequences are simplified in the GUI-based tool. There are no requirements for raw data conversion. Reference sequences can be downloaded from public databases and used without further manipulation. Automated format conversion is also available.

### High-throughput ability

Parallel processing in eRNA allows for the analysis of multiple RNA samples at the same time. This approach efficiently uses computation power by balancing computer performance and running time. The sample management function exempts biological researchers from manually inputting numerous data sets. eRNA can also be used for both small- and large-scale RNA-seq data. The package has been successfully tested in a personal computer as well as in an advanced server computer. Biological researchers may customize their own computer platforms at a relatively low cost.

### Integration

eRNA is aiming at helping users gain insight into the underlying biology of the expressed RNAs determined by RNA-seq. The current version of eRNA has been integrated with the other tools for identification, differential expression profiling analysis, and visualization of known miRNAs and mRNAs, as well as the discovery of novel miRNAs, target gene screening using recursive partitioning analysis, sequence alignment, and the visualization of sequencing quality control. Additional mRNA-seq tools [31,32] besides the TopHat-Cufflinks pipeline used in the module “mRNA identification”, more differential gene expression methods [33] besides the R package DEseq used in the module “Targets screening”, and the enrichment tools on pathway analysis [34] will be incorporated into future versions of eRNA.

## Conclusions

eRNA can be used for the identification of RNAs and expression profiling analysis of miRNA-seq and mRNA-seq data. It is easy to use and requires no prior specific computer science knowledge. A user-friendly framework allows biological researchers to focus on biological interpretation. Parameter settings and preparations of raw data and reference sequences are simplified. Parallel processing in eRNA allows for the analysis of multiple RNA samples at the same time. The sample management function exempts biological researchers from manually inputting numerous data sets.

## Availability and requirements

eRNA is available for free download and use at https://sourceforge.net/projects/erna/?source=directory according to the GNU Public License. The user manual including its installation and the required running environments is also included in the eRNA package. Any use by non-academics requires license. We developed eRNA using Perl language programming in the Linux operating system. The developing and testing environments were Fedora Linux 17 (X_86 64 bits) in a personal computer equipped with one Intel Core i7-3770 K CPU (3.5 GHz, 4 cores per CPU) and 32 GB memory and a Red Hat Enterprise Linux Server (release 5.9, X_86 64 bits) equipped with four Intel Xeon X5687 CPUs (3.6 GHz, 4 cores per CPU) and 96 GB memory. Other software environments included Perl (version 5.14), Perl-Gtk2 (version 1.241), Bioperl (version 1.6, http://www.bioperl.org/), and R (version 2.15, http://www.r-project.org/).

## Abbreviations

GUI: Graphic user interface; HTS: High-throughput sequencing; miRNA: microRNA; miRNA-seq: microRNA sequencing, mRNA-seq, mRNA sequencing; RNA-seq: RNA sequencing.

## Competing interests

The authors declare that they have no competing interests.

## Authors’ contributions

TZY developed the software and wrote the manuscript. LW supervised the approach. XYH prepared RNA seq libraries. MK, SNT, and LB identified biological case studies. LW, RLD, XYH, MJD, MK, LB, and SNT revised the final manuscript. All authors read and approved the final manuscript.

## Supplementary Material

Additional file 1: Table S1 Comparison of the pipelines on the identification of miRNAs. **Table S2** Comparison of the open-source pipelines on the identification of mRNAs. Click here for file
