# Supplementary material for: eRNA: a graphic user interface-based tool optimized for large data analysis from high-throughput RNA sequencing
Source: BMC Genomics. 2014 Mar 5;15:176. doi: 10.1186/1471-2164-15-176 (PMC4029068; doi:10.1186/1471-2164-15-176)
Supplement: Additional file 1: Table S1 — Comparison of the pipelines on the identification of miRNAs. Table S2 Comparison of the open-source pipelines on the identification of mRNAs. [file 1471-2164-15-176-S1.DOC]

**Additional file 1: Table S**1. Comparison of the pipelines on the identification of miRNAs

| **Pipelines** | eRNA | Galaxy | miRDeep2 | miRspring | DSAP | mirTools | miRanalyzer | miRExpress | SeqBuster | miRNAkey | E-miR | MIReNA |
| --- | --- | --- | --- | --- | --- | --- | --- | --- | --- | --- | --- | --- |
| **Platform** | Standalone | Standalone/web-based | Standalone | Standalone | Web-based | Web-based | Standalone/web-based | Standalone | Standalone | Standalone | Standalone | Standalone |
| **Running environment** | Linux | No limits | Linux | Linux | No limits | No limits | No limits | Linux | Linux or Mac OS X | Linux or Mac OS X | Linux | Linux |
| **User interface** | GUIs or command-line | GUIs | Command-line | Command-line | GUIs | GUIs | GUIs | Command-line | GUIs | Command-line | Command-line | Command-line |
| **Method of sequence mapping** | Bowtie1 used | Bowtie1, Bowtie2, or BWA used | Bowtie1 used | Bowtie1 used | House-keeping algorithm | SOAP used | Bowtie1 used | Single Instruction Multiple Data (SIMD) instructions | Blast used | BWA used | Bowtie1or Eland used | house-keeping algorithm |
| **Method of miRNA expression profiling analysis** | R packages DEseq used | No | No | No | No | Bayesian | R packages DEseq used | No | House-keeping algorithm | SEQ-EM algorithm | No | No |
| **Reference limits** | known miRNA database | Genome sequences and known miRNA database | Genome sequences and known miRNA database | Genome sequences and known miRNA database | Genome sequences and known miRNA database | Genome sequences and known miRNA database | Genome sequences and known miRNA database | Known miRNA database | Known miRNA database | Known miRNA database | Genome sequences and known miRNA database | known miRNA database |
| **Batch data processing** | Yes | No | No | No | No | No | No | No | No | No | No | No |
| **FASTQ support** | Yes | Yes | No | No | No | No | Yes | Yes | Yes | Yes | Yes | No |
| **Sample management** | Yes | No | No | No | No | No | No | No | No | No | No | No |
| **Parallel processing** | Yes | Unknown | No | No | Unknown | Unknown | No | No | No | No | No | No |

**Additional file 1: Table S2. Comparison of the open-source** pipelines on the identification of mRNAs

| **Pipelines** | eRNA | Galaxy^1^ | RNA-seq Toolkit | ArrayExpressHTS^2^ | Chipster | GENE-Counter | ExpressionPlot | GenePattern | GeneProf | RobiNA | TCW |
| --- | --- | --- | --- | --- | --- | --- | --- | --- | --- | --- | --- |
| **Platform** | Standalone | Standalone | Standalone | Standalone | Standalone | Standalone | Standalone | Standalone/web-based | Web-based | Standalone | Standalone |
| **Running environment** | Linux | Linux | Linux | Linux/Mac OS X | No limits | Linux | Linux | Linux/Windows/Mac OS X | Linux/Windows/Mac OS X | Linux/Windows/Mac OS X | Linux |
| **Programming environment** | Perl | Python | Shell script | R | Java | Perl | VirtualBox, Perl | Java | Unknown | Java | Java |
| **User interface** | GUIs or command-line | GUIs | Command-line | GUIs | GUIs | Command-line | Command-line | GUIs | GUIs | GUIs | GUIs |
| **Method of genome mapping** | TopHat and Bowtie | TopHat and Bowtie | TopHat and Bowtie | BWA, TopHat and Bowtie | BWA, TopHat and Bowtie | TopHat, SOAP2 and Bowtie | Bowtie | BWA, TopHat and Bowtie | Bowtie, Picard, TopHat | Bowtie | Blast |
| **Method of transcripts assembling** | Cufflinks | Cufflinks | Cufflinks | Cufflinks and MMSEQ | Cufflinks | Cufflinks | Splice junction databases | Cufflinks | Cufflinks | Unknown | CAP3 |
| **Method of differential expression analysis** | R package : Cuffdiff or DEseq | R package : Cuffdiff | R package : Cuffdiff | R package : Cuffdiff, DEseq, or edgeR | R package : Cuffdiff, DEseq, or edgeR | R package : DEseq, or edgeR | R package : DEseq | R package : Cuffdiff, | R package : DEseq, or edgeR | R package : DEseq, or edgeR | R package : DEseq, or edgeR |
| **Data management** | A sample management tool | No | No | No | No | No | No | Unknown | A sample annotation tool | A experiment designer | MySQL database |
| **Parallel processing** | Multi-threads^3^ | Multi-threads^3^ | Multi-threads^3^ | R cloud | Unknown | No | No | Unknown | Unknown | No | Clusters |

1. Galaxy can be accessed through the web site.

2. ArrayExpressHTS can be accessed the EBI R cloud.

3. Bowtie, TopHat and Cufflinks provide multi-threading analysis.
